# Cell studies of hybridized carbon nanofibers containing bioactive glass nanoparticles using bone mesenchymal stromal cells

**DOI:** 10.1038/srep38685

**Published:** 2016-12-07

**Authors:** Xiu-Rui Zhang, Xiao-Qing Hu, Xiao-Long Jia, Li-Ka Yang, Qing-Yang Meng, Yuan-Yuan Shi, Zheng-Zheng Zhang, Qing Cai, Yin-Fang Ao, Xiao-Ping Yang

**Affiliations:** 1 State Key Laboratory of Organic-Inorganic Composites; Beijing Laboratory of Biomedical Materials; Beijing University of Chemical Technology, Beijing 100029, P. R. China; 2 Institute of Sports Medicine, Beijing Key Laboratory of Sports Injury, Peking University Third Hospital, Beijing 100191, P. R. China

## Abstract

Bone regeneration required suitable scaffolding materials to support the proliferation and osteogenic differentiation of bone-related cells. In this study, a kind of hybridized nanofibrous scaffold material (CNF/BG) was prepared by incorporating bioactive glass (BG) nanoparticles into carbon nanofibers (CNF) via the combination of BG sol-gel and polyacrylonitrile (PAN) electrospinning, followed by carbonization. Three types (49 s, 68 s and 86 s) of BG nanoparticles were incorporated. To understand the mechanism of CNF/BG hybrids exerting osteogenic effects, bone marrow mesenchymal stromal cells (BMSCs) were cultured directly on these hybrids (contact culture) or cultured in transwell chambers in the presence of these materials (non-contact culture). The contributions of ion release and contact effect on cell proliferation and osteogenic differentiation were able to be correlated. It was found that the ionic dissolution products had limited effect on cell proliferation, while they were able to enhance osteogenic differentiation of BMSCs in comparison with pure CNF. Differently, the proliferation and osteogenic differentiation were both significantly promoted in the contact culture. In both cases, CNF/BG(68 s) showed the strongest ability in influencing cell behaviors due to its fastest release rate of soluble silicium-relating ions. The synergistic effect of CNF and BG would make CNF/BG hybrids promising substrates for bone repairing.

Repairing of bone defects remains a challenge in clinical therapy. In bone tissue engineering, the artificial extracellular environment should support the proliferation and osteogenic differentiation of bone-related cells such as osteoblasts and bone marrow mesenchymal stromal cells (BMSCs)^1^,^2^. For this critical requirement, strategies to build cell scaffolds for bone regeneration have applied the concept of mimicking natural extracellular matrix (ECM) by using nanocomposite biomaterials^3^,^4^,^5^,^6^. From numerous studies, nanofibrous scaffolds containing osteo-inductive components have shown advantages over other biomaterials in enhancing bone regeneration^7^,^8^,^9^,^10^.

Among nanofibrous biomaterials, carbon nanofibers (CNF) have attracted great interest in the field of bone regeneration in considering their excellent mechanical strength, unique magnetic and electric properties^11^. Moreover, it was reported that carbon nanomaterials including CNF were able to promote attachment and proliferation of bone-related cells^12^,^13^,^14^,^15^,^16^. To improve the ability of CNF in inducing osteogenic differentiation, some researches electrospun the mixture solution of polyacrylonitrile (PAN) and hydroxyapaptite (HA) nanoparticles, followed by stabilization and carbonization^17^. However, a popular problem with this approach was hard to achieve the homogeneous dispersion of HA nanoparticles in the final CNF. To produce hybridized CNF containing homogeneously distributed ceramic nanoparticles, a combination of ceramic precursor sol-gel and PAN electrospinning with subsequent stabilization and carbonization has been well developed^18^,^19^. Reasonably, in our previous studies, bioceramic components including calcium phosphate (CaP) and bioactive glass (BG) had been incorporated into CNF by a similar way^20^,^21^,^22^,^23^. Compared to pure CNF, it was found CaP (CNF/CaP) or BG nanoparticle loaded CNF (CNF/BG) was able to accelerate apatite deposition in simulated body fluid (SBF) and to improve cell affinity, showing strong dependence on the chemical composition, morphology and crystalline structure of ceramic nanoparticles^21^,^23^. Basically, the enhancements were ascribed to the dissolution behaviors of CaP or BG components^24^,^25^,^26^,^27^, which endowed the hybridized CNF higher bioactivity than pure CNF. However, no further detailed study has been carried out to look into the effects of hybridized CNF on osteogenic differentiation of BMSCs and the mechanism behind these effects. It is interesting to know which factor, the CNF itself or the incorporated ceramic component, playing the dominant role in regulating the osteogenic effect of hybridized CNF.

Herein, CNF/BG hybrids were prepared by incorporating BG components of three different chemical compositions, and used for the study to induce *in vitro* osteogenic differentiation of BMSCs. BG could provide calcium, phosphorus and silicic ions when it was exposed to body fluid or culture medium^28^. The bioactivity of BG was closely related to its dissolution behavior^29^,^30^, while the amounts of released ions depended on its chemical compositions^31^,^32^. Therefore, BG was chosen as a proper model ceramic component in producing hybridized CNF in this study. BMSCs were cultured in two manners, in which, they were seeded onto CNF/BG hybrids directly, or they were cultured in transwell chamber containing CNF/BG hybrids but not contacting the materials. In the former culture manner, cell proliferation and differentiation will be affected by both the CNFs and the BG component. While in the latter culture manner, it targets to focus on the effect of dissoluble ingredients from CNF/BG hybrids on the biological behaviors of BMSCs without considering the possible influences from features such as morphology and roughness etc. In both the cases, cell proliferation and osteogenic differentiation were evaluated. Quantitative real-time PCR (qRT-PCR) and enzyme-linked immuno sorbent assay (ELISA) were applied to measure the expressions of osteogenic differentiation related genes and markers. At the same time, the ion release behaviors from CNF/BG hybrids were determined. Thus, the contributions of the soluble ingredients and the contact effect of CNF/BG hybrids on cell proliferation and osteogenic differentiation were able to be correlated, respectively.

## Results

### Prepared CNF/BG hybrids

**Figure 1:**
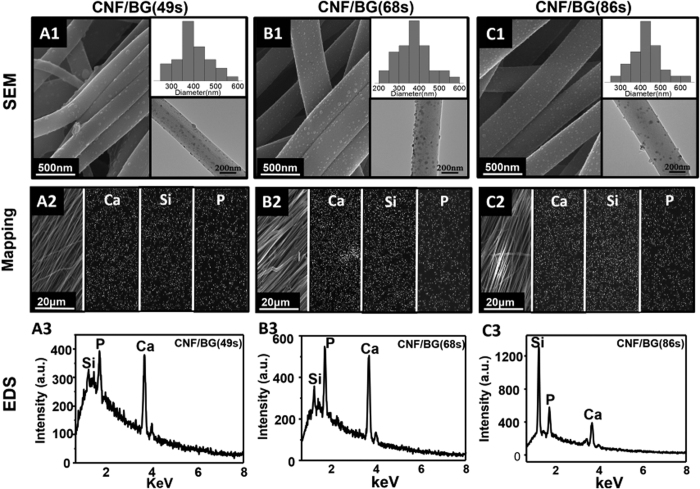
Characterizations of prepared CNF/BG hybrids by using SEM observation (A1, B1, C1), element mapping (A2, B2, C2) and EDS analysis (A3, B3, C3): (A1–A3) CNF/BG (49 s); (B1–B3) CNF/BG (68 s); (C1–C3) CNF/BG (86 s). The insets in SEM images are fiber diameter distribution and TEM image of the corresponding CNF/BG hybrid.

**Figure 2:**
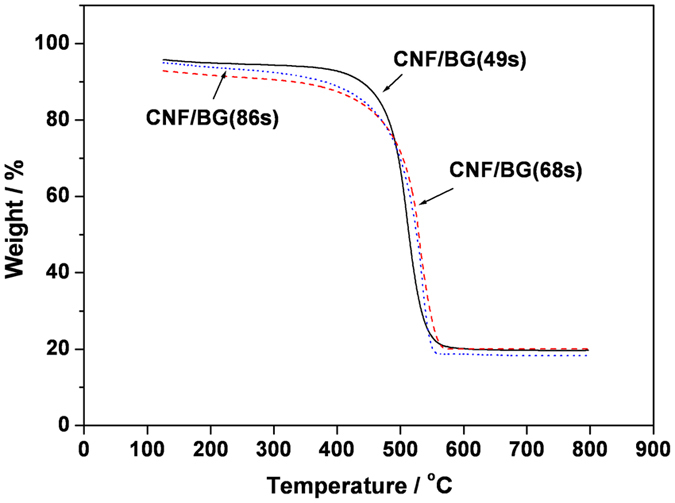
TGA curves of prepared CNF/BG hybrids.

Morphology and element analysis of prepared CNF/BG hybrids are shown in Fig. 1. After carbonization at 1000 °C for 1 h in N_2_ atmosphere, the electrospun PAN nanofibers transformed into CNFs due to dehydrogen and denitrogen reactions upon thermal treatment. At the same time, the sol-gel precursors transformed into BG component during the sintering. As shown in Fig. 1, the resulted CNF/BG hybrids were continuous bead-free nanofibers with uniformly decorated nanoparticles. The nanofibers displayed parallel alignment due to the use of rolling rod as the collector in electrospinning (Fig. 1A1–C1). For all the three hybrids, the average fiber diameter was ~400 nm and the average size of nanoparticles was ~10 nm. As those element mapping images shown (Fig. 1A2–C2), characteristic elements of BG component including Ca, Si and P were all detected. Thus the nanoparticles were identified BG nanoparticles and their homogeneous distribution along the fibers was a result of being expelled from the dense graphite carbon structure of CNF^22^. Confirmed by the EDS results (Fig. 1A3–C3), the contents of Ca, Si and P elements were different in the three CNF/BG hybrids that the strengths of corresponding signals were in accordance with those feeding ratios presented in Table 1. In other words, the three CNF/BG hybrids contained different types of BG and the content of Si element was in the order of CNF/BG(49 s) < CNF/BG(68 s) < CNF/BG(86 s). Whereas, the contents of BG component in all the three CNF/BG hybrids were almost the same (~20 wt.%), as those TGA curves shown in Fig. 2, showing independence on the type of BG.

### Ion release from CNF/BG hybrids

**Figure 3:**
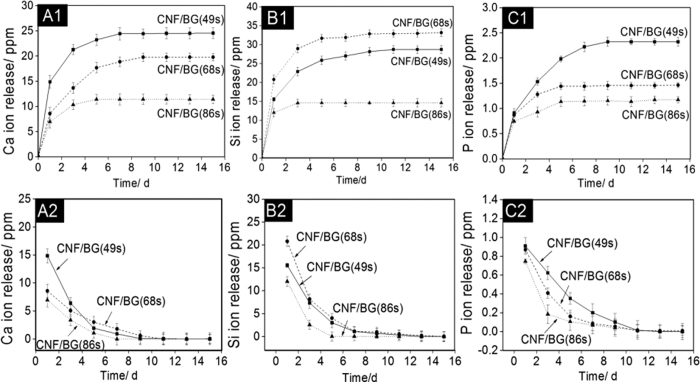
Release of different ions from various CNF/BG hybrids along with soaking time in deionized water at 37 °C: (A1–C1) the accumulative release curves; (A2–C2) the change in the real time ion concentration at each time point with refreshing deionized water every other day.

When being exposed to water, those BG nanoparticles in CNF/BG hybrids would dissolve to release ions like Ca^2+^, SiO_3_^2−^ and PO_4_^3−^. However, the release behaviors were closely related to the type of BG component. From the accumulative release profiles (Fig. 3A1–C1), on the whole, all the ions displayed a fast and continuous release from all the three CNF/BG hybrids within the first week, then leveled off along with longer soaking time. In both the release of calcium and phosphorus ions, CNF/BG(49 s) demonstrated the fastest release rate, followed by CNF/BG(68 s) and CNF/BG(86 s). While in the release of silicic ion, it was CNF/BG(68 s) showing the fastest release rate followed by CNF/BG(49 s) and CNF/BG(86 s). In comparing the accumulative released amounts of different ions from different CNF/BG hybrids, together with the fact that the contents of BG components in all the CNF/BG hybrids were similar (Fig. 2), it was inferred that the solubility of BG(86 s) was apparently lower than those of BG(49 s) and BG(68 s).

In Fig. 3A2–C2, the ion release behaviors were presented by plotting the real time ion concentration as function of soaking time. As described in the experimental section, the release medium was refreshed every other day to mimic the cell culture condition. Therefore, from Fig. 3A2–C2, it was intended to reveal the changes in ion concentrations along with the regularly refreshing of culture medium during cell culture. It could be seen that the dissolution of BG component from the CNF/BG hybrids was quite fast, and the released ion concentrations reached the maximum values within 1 day. Along with the medium being refreshed every 2 days, the released ion concentrations in the media decreased gradually. When the soaking time was longer than 10 days, almost no ion could be detected in the released media in all the cases.

### Cell viability

**Figure 4:**
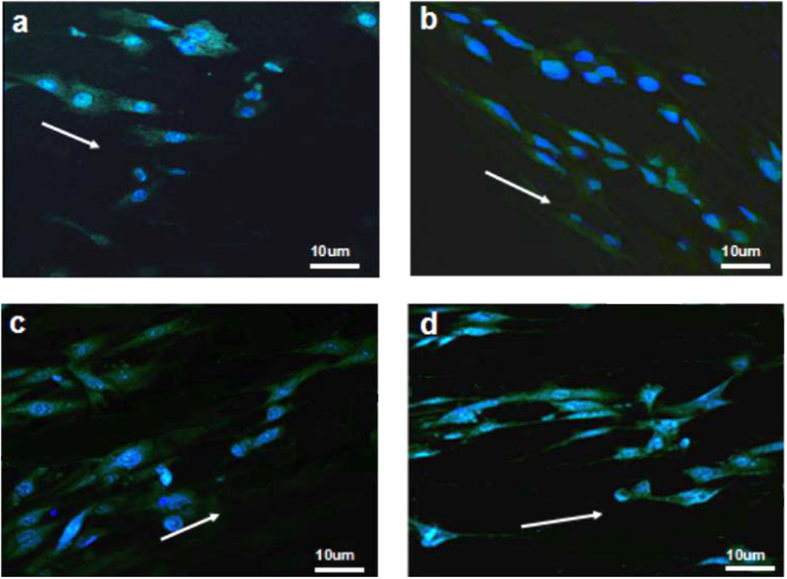
Fluorescence observation on morphology of BMSCs cultured on various CNF/BG hybrids and pure CNF for 1 day: (**a**) CNF/BG(49 s); (**b**) CNF/BG(68 s); (**c**) CNF/BG(86 s); (**d**) pure CNF. Green sections indicate cell cytoskeleton and blue sections indicate cell nucleus, and the white arrows indicate the orientation of nanofibers.

To evaluate biocompatibility and cell affinity of the prepared CNF/BG hybrids and pure CNF, firstly, BMSCs were seeded onto the substrates and cultured 1 day allowing for cell attachment and morphology observation. From Fig. 4, BMSCs on all substrates were in spindle-like shape with oval nucleus, which was the normal morphology for spreading BMSCs. The green color represented the cell cytoskeleton and the blue color displayed the cell nucleus, because a green fluorescent protein (GFP) transfected BMSCs were applied in this study, and the nucleus was stained with Hoechest 33258. Due to the parallel-alignment of CNF (Fig. 1), the cells were elongated along fiber direction because of contact guide effect. These facts indicated that BMSCs could adhere and spread well on both pure CNF and CNF/BG hybrids.

**Figure 5:**
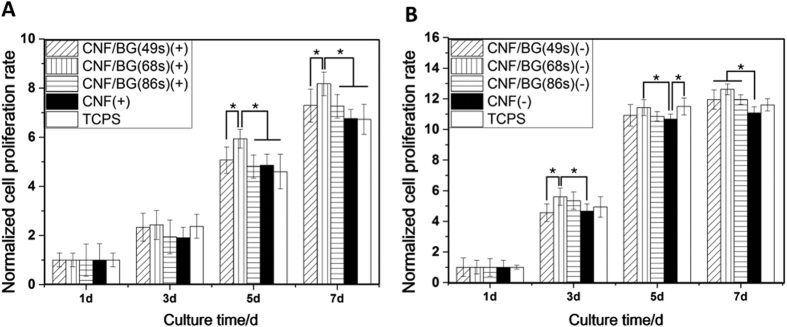
Proliferation of BMSCs on various CNF/BG hybrids in comparison with pure CNF and TCPS by using Alamar blue assay in different culture manners: (**A**) BMSCs cultured directly on materials; (**B**) BMSCs cultured in Transwell in the presence of materials but not contacting the materials. The cell growth rate is normalized to the first day data. *p ≤ 0.05, significant.

Then cell proliferation was assessed in both the contact and the non-contact culture manners by Alamar blue assay. The cell growth rate was normalized to the first day data and shown in Fig. 5. In all the cases, BMSCs were seen able to proliferate continuously with culture time; however, their growth rates differed and closely depended on both the feature of the substrate and the manner of cell culture. When BMSCs were cultured directly on the materials (Fig. 5A), it was found that BMSCs proliferated significantly faster on CNF/BG hybrids than on both pure CNF and TCPS, while cell growth rates were comparable between the pure CNF and the control. The incorporation of BG component made the growth rate of BMSCs being faster on CNF/BG hybrids than on pure CNF, especially when 68 S type BG component was incorporated. When in the case of non-contact culture (Fig. 5B), similarly, BMSCs also proliferated slightly faster in the case of CNF/BG hybrids than in the case of control, while the cell proliferation rate was the slowest in the presence of pure CNF. Still, it was CNF/BG(68 s) displaying the strongest promotion in cell growth. In the non-contact culture condition, however, the difference between different groups was not so significant as those in the contact culture condition. Interestingly, the observations in Fig. 5A and B suggested that the contact effect and the released ions both played roles in regulating cell growth. Another thing noticeable here was that the relative cell proliferation rate was faster in the non-contact culture manner using 24-well transwell plate than in the contact culture manner using 96-well plate, whether it contained materials or not. During cell culture, it was found the GFP-transfected BMSCs demonstrated fast growth rate even at low seeding density (500 cell/well). Therefore, it was suggested the 24-well transwell culture condition was able to provide more abundant nutrients to support the fast growth of BMSCs in comparison with the 96-well plate in contact culture.

**Figure 6:**
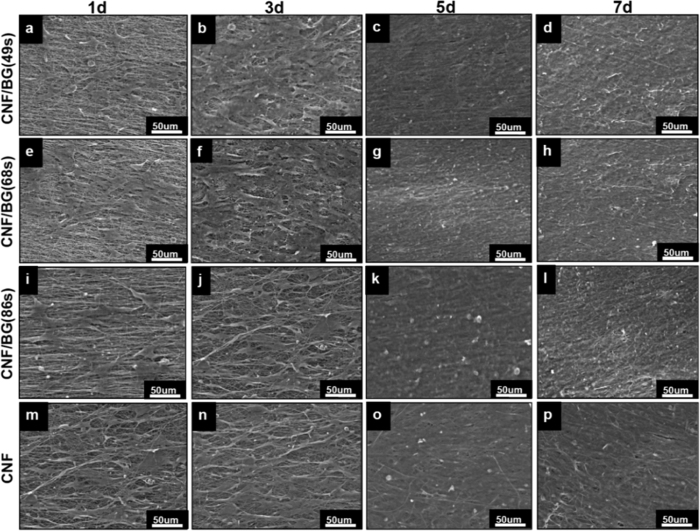
SEM observations on morphology and proliferation of BMSCs cultured on different CNF/BG hybrids and pure CNF for different times.

After being cultured on the materials for 1, 3, 5 and 7 days in the contact manner, the cellular constructs were collected for SEM observation. As shown in Fig. 6, BMSCs adhered firmly onto pure CNF and CNF/BG hybrids within 1 day, and spread into spindly shape along the direction of nanofibers. With longer culture time, the cell numbers on all the substrates increased and a large amount of ECM was excreted. To the fifth day of cell culture, the nanofibrous substrates had been fully covered with confluent cells. The cell morphology was normal on all these CNF-based substrates, showing no obvious difference between groups.

### Osteogenic differentiation

**Figure 7:**
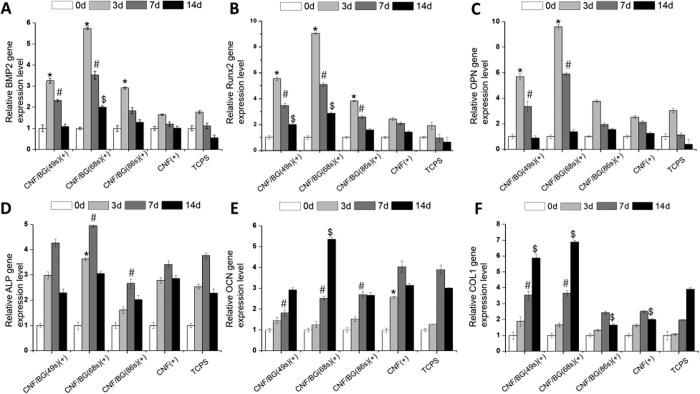
Relative expressions of osteogenic differentiation related genes including BMP2 (**A**), Runx2 (**B**), OPN (**C**), ALP (**D**), OCN (**E**) and Col I (**F**) of BMSCs being cultured for 0, 3, 7 and 14 days on various CNF/BG hybrids and pure CNF using TCPS as control. (*) Significant difference compared to TCPS on day 3 (p < 0.05); (#) Significant difference compared to TCPS on day 7 (p < 0.05); ($) Significant difference compared to TCPS on day 14 (p < 0.05).

**Figure 8:**
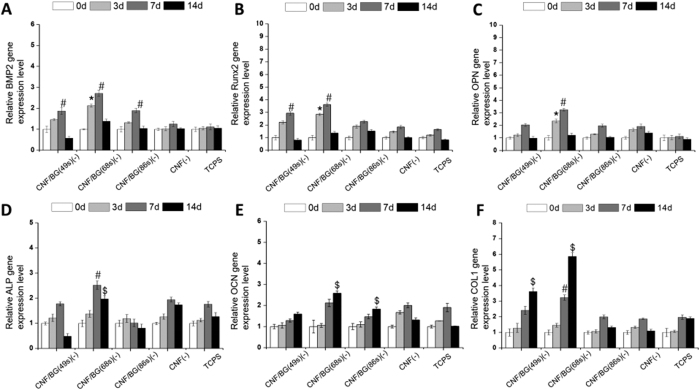
Relative expressions of osteogenic differentiation related genes including BMP2 (**A**), Runx2 (**B**), OPN (**C**), ALP (**D**), OCN (**E**) and Col I (**F**) of BMSCs being cultured in Transwell for 0, 3, 7 and 14 days in the presence of various CNF/BG hybrids and pure CNF using TCPS as control. (*) Significant difference compared to TCPS on day 3 (p < 0.05); (#) Significant difference compared to TCPS on day 7 (p < 0.05); ($) Significant difference compared to TCPS on day 14 (p < 0.05).

Osteogenic differentiation of BMSCs were also evaluated in both contact and non-contact culture manners by quantitatively analyzing expressions of related genes including BMP2, Runx2, OPN, ALP, OCN and Col I. The results are presented in Figs 7 and 8, respectively. All the gene expression data were normalized to house-keeping 18 S, and all the data were normalized to the original value (i.e. the data of 0 day) of corresponding samples.

When BMSCs were cultured directly on materials, as shown in Fig. 7, the osteogenic genes were up-regulated with culture time in different trends. On all substrates including the TCPS control, expressions of BMP2, Runx2 and OPN reached the maximum value at the third day, expression of ALP reached the maximum at day 7 of cell culture, while both OCN and Col I demonstrated the highest expression at day 14 of cell culture. After the point of the highest expression, all the genes were down-regulated with longer culture time. These results were in accordance with the known facts that the six genes were related to different stages of osteogenic differentiation. On the other hand, the gene expressions of BMSCs cultured on both CNF/BG(86 s) and pure CNF did not show significant difference to those on TCPS. While the osteogenic differentiation of BMSCs was significantly enhanced on CNF/BG(49 s) and CNF/BG(68 s) as confirmed by their higher expressions of osteogenic genes. Noticeably, the CNF/BG(68 s) had the strongest ability in enhancing osteogenic differentiation among all the substrates. In comparison with the case of TCPS, the expressions of BMP2 (5.8-fold), Runx2 (9-fold), OPN (9.6-fold), ALP (5-fold), OCN (5.3-fold) and Col I (6.9-fold) were all substantially improved in the case of CNF/BG(68 s).

When in the case of non-contact culture (Fig. 8), the up-regulation of the six genes with culture time changed in a similar way to those in the case of contact culture. Still, it was the group of CNF/BG(68 s) displaying the highest gene expressions, followed by the group of CNF/BG(49 s). And both of them had stronger ability in enhancing osteogenic differentiation than other groups. Except the expression of BMP2, expressions of other five genes in the group of CNF/BG(86 s) demonstrated comparable results with the case of TCPS or in the presence of pure CNF. These findings suggested that those released ions from CNF/BG hybrids into the media were able to enhance osteogenic differentiation, but depending on the amounts of released ions.

**Figure 9:**
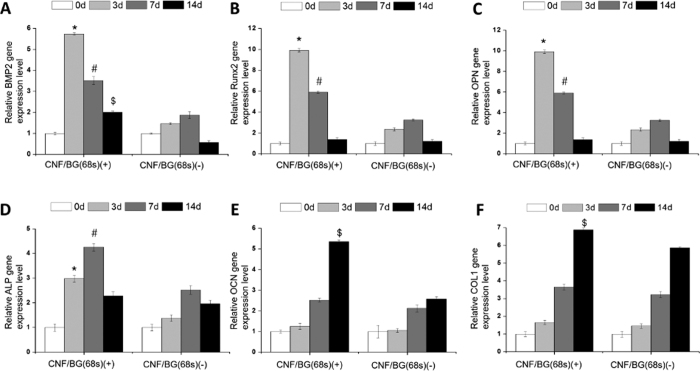
Comparison of relative gene expressions of osteogenic differentiation related genes including BMP2 (**A**), Runx2 (**B**), OPN (**C**), ALP (**D**), OCN (**E**) and Col I (**F**) of BMSCs being cultured on CNF/BG(68 s) directly and in Transwell in the presence of CNF/BG(68 s).

In comparing Figs 7 and 8, one minor and one major difference were able to be identified in gene expressions between the two culture manners. The minor difference was that the highest expressions of BMP2, Runx2 and OPN were achieved at the seventh day in the non-contact culture instead of the 3 days in the contact culture. The major difference was that the up-regulating degrees in gene expressions were much higher in the case of contact culture than in the case of non-contact culture, when all the data were normalized by using the TCPS results. For clarity, the comparisons in gene expressions between the two culture manners are illustrated in Fig. 9, taking CNF/BG(68 s) as the example. From the figure, apparently, the contact culture on CNF/BG(68 s) could promote osteogenic differentiation of BMSCs more efficiently than the non-contact culture manner, that the gene expressions in the former case were almost 2-fold higher than those in the latter case. This finding suggested that the surface feature and the contact effect of substrates would play more important roles in regulating cell biological behaviors than soluble factors.

**Figure 10:**
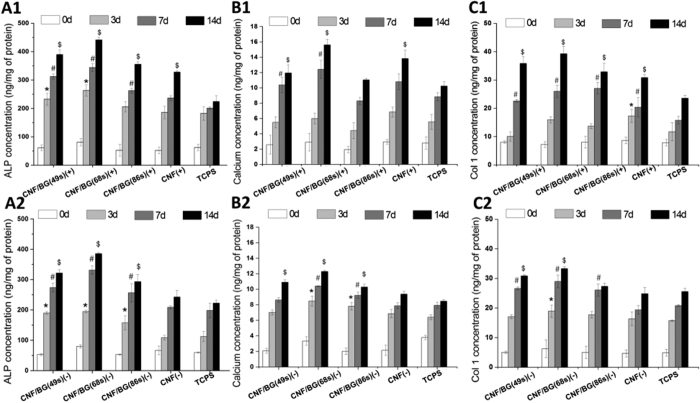
ELISA assay on ALP activity, calcium deposition and Col I synthesis of BMSCs being cultured on various CNF/BG hybrids directly (A1, B1, C1) and in Transwell in the presence of corresponding CNF/BG hybrid (A2, B2, C2) for 3, 7, 14 days using pure CNF and TCPS as controls. (*) Significant difference compared to TCPS on day 3 (p < 0.05); (#) Significant difference compared to TCPS on day 7 (p < 0.05); ($) Significant difference compared to TCPS on day 14 (p < 0.05).

To confirm the effects of CNF/BG hybrids on osteogenic differentiation of BMSCs, ELISA assays were also performed in the two culture manners to determine ALP activity, calcium deposition and Col I synthesis along with culture time (Fig. 10). Whether in the contact (Fig. 10A1–C1) or the non-contact culture (Fig. 10A2–C2), in a whole, the produced levels of these osteogenic differentiation markers increased continuously with culture time in all the cases. In comparison with TCPS, osteogenic differentiation of BMSCs cultured in the cases with materials being present were all promoted in both the culture manners. And all the groups containing CNF/BG hybrids demonstrated stronger ability in enhancing osteogenic differentiation of BMSCs than the group containing only pure CNF. Among the three CNF/BG hybrids, the enhancing effect could be seen in the order of CNF/BG(68 s) > CNF/BG(49 s) > CNF/BG hybrids(86 s) in both the contact and the non-contact culture. For each osteogenic differentiation marker, besides, the values could be seen higher in the case of contact culture than in the case of non-contact culture for each corresponding material. These findings were well in accordance with the aforementioned qRT-PCR results.

### Gene down-regulation by inhibition of BMP activity

**Figure 11:**
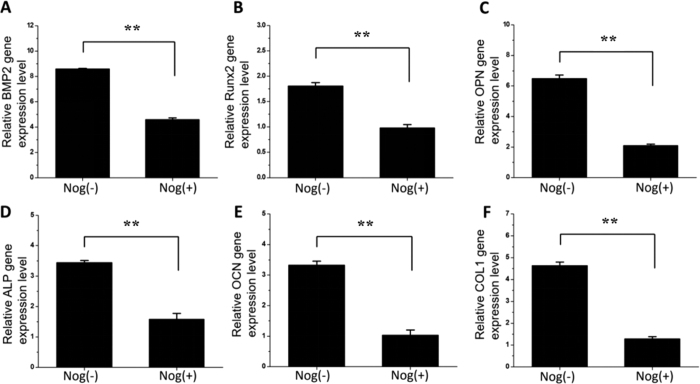
Comparisons between relative expressions of osteogenic genes in the cases of BMSCs being cultured on CNF/BG(68 s) for 3 days without or with the use of BMP specific inhibitor (noggin) in the cell culture: (**A**) BMP2; (**B**) Runx2; (**C**) OPN; (**D**) ALP; (**E**) OCN; (**F**) Col I. **p < 0.01, highly significant.

As above found, CNF/BG(68 s) was biocompatible and able to promote osteogenic differentiation of BMSCs the most efficient among all the materials. The expressions of the six BMP signaling pathway related genes were significantly enhanced by CNF/BG(68 s). If so, conversely, these genes would be down-regulated if the BMP signaling pathway was inhibited. The specific inhibitor noggin was used to inhibit BMP activity. In comparison with the normally cultured BMSCs on CNF/BG(68 s), as shown in Fig. 11A, the addition of noggin decreased the expression of BMP2 significantly. The expression of Runx2, a downstream regulator in the Wnt/BMP signaling pathway, was also down-regulated accordingly (Fig. 11B). Similar down-regulations were detected for other four genes (OPN, ALP, OCN and Col I) (Fig. 11C–F), indicating the osteogenic differentiation of BMSCs was indeed significantly inhibited in the presence of BMP signaling pathway inhibitor noggin.

## Discussion

Cell proliferation and differentiation were influenced by many factors including material composition, topographical cue and soluble components^23^,^24^,^33^,^34^. For the prepared CNF/BG hybrids and pure CNF in this study, they were nanofibrous constructs, which favored the cell attachment and proliferation because they mimicked the three dimensional structure of natural ECM^35^. Therefore, from Fig. 5A, BMSCs cultured on pure CNF proliferated faster than those on flat TCPS in the contact culture. Besides, it was reported that nano-scaled carbon materials demonstrated a strong ability of absorbing proteins because the π electron cloud of graphite structure could intact with the hydrophobic part of protein^36^. The carbon nanomaterials could specifically absorb the proteins relating to cell proliferation, such as albumin and fibronectin^37^. Moreover, the incorporation of BG nanoparticles into the CNF had increased the surface roughness of nanofibers in CNF/BG hybrids, as shown in Fig. 1. It has been well known that substrates with microroughness could enhance cell attachment and promote cell growth in comparison with smooth-surfaced substrates^38^,^39^,^40^. Accordingly, BMSCs seeded on all the three CNF/BG hybrids demonstrated faster proliferation rates than those cells on pure CNF (Fig. 5A). Difference was also found in the cell proliferation rates among the three CNF/BG hybrids, however, the explanation might be ascribed to reasons other than fiber morphology.

From Figs 1 and 2, the three CNF/BG hybrids clearly demonstrated similar morphology and similar content of BG component. The main difference between these CNF/BG hybrids was the chemical compositions of their incorporated BG components. And from Fig. 3, it was identified that ions release from these CNF/BG hybrids were significantly different, showing dependence on chemical compositions of BG component. In the non-contact culture, cell proliferation could be only related to the released ions without considering the fiber morphology when the systems contained CNF/BG hybrids. As shown in Fig. 5B, the proliferation rates of BMSCs were slightly faster in the presence of CNF/BG hybrids in comparison with the group containing pure CNF and the control group. The group of CNF/BG(68 s) demonstrated the fastest cell proliferation rate among the three CNF/BG hybrids. In comparison with the ion release results of Fig. 3, it could be seen the trend of BMSCs proliferation in non-contact culture were in accordance with the released amounts of silicium component when the system contained different CNF/BG hybrids. These findings suggested that the released ions from CNF/BG hybrids, being diluted in culture medium, had some but not so significant effects on cell proliferation.

Nevertheless, the BMSCs cultured directly on CNF/BG(68 s) displayed significantly higher growth rate than those cells on both CNF/BG(49 s) and CNF/BG(86 s) (Fig. 5A), showing different trend from those cases in the non-contact culture. The most possible explanation for this phenomenon was thought that the fiber morphology and the released ions had played a synergistic effect on the proliferation of BMSCs. Cells attached fast and well onto CNF/BG hybrids due to the BG nanoparticle decorated fiber morphology. Cells proliferated well due to the ions nearby supplied by the fast dissolution of BG component. And it was inferred that the CNF/BG(68 s) could provide the most abundant soluble silicium ions among the three CNF/BG hybrids, which caused BMSCs to proliferate the fastest on CNF/BG(68 s). In other words, the effect of silicium component on cell proliferation was more efficient than calcium and phosphorus components^41^.

About the cell proliferation in the non-contact culture (Fig. 5B), noticeably, the growth rate of BMSCs was slower in the presence of pure CNF than in the group of TCPS, although the pure CNF could not release any ions. Since BMSCs could spread and proliferate well with rich ECM being excreted on all the CNF-based materials (Figs 5A and 6), these CNF-based materials were identified to be non-cytotoxic. In publications, the absorption capacity of carbon nanomaterials had been reported^42^. In evaluating BMSC proliferation by Alamar blue assay, part of the formed fluorescent products might be absorbed by the CNF-based materials, and phenomenally the cells grew slower in the presence of pure CNF than the control in the non-contact culture. In the case of CNF/BG hybrids, although this adsorbing effect still existed, the enhancements of released ions on cell growth had surpassed this adverse effect. In the contact culture, the enhancements of CNF/BG hybrids on cell proliferation were even more significant due to the synergetic effect of both nanofibrous morphology and bioactive BG component.

As for osteogenic differentiation, in the non-contact culture, the observations were totaly different to the cell proliferation. As shown in Figs 8 and 10A2–C2, both the gene expressions and the ELISA results revealed that the osteogenic differentiation of BMSCs was enhanced in the presence of CNF/BG hybrids in comparison with the control and the CNF group. Correlating to their release behaviors, the CNF/BG(68 s) demonstrated the strongest ability in promoting osteogenic differentiation due to its highest amounts of released silicium ions into the culture medium, followed by CNF/BG(49 s) and CNF/BG(86 s). No significant difference in osteogenic differentiation could be identified between the control and the CNF group. These finding strongly proven that the bioactivity of CNF/BG hybrids came from the dissolution of BG component and closely depended on the chemical compositions of BG component, which decided the ion release behaviors.

When BMSCs were directly cultured on these CNF/BG hybrids, from Figs 7 and 10A1–C1), it could be seen that the osteogenic differentiation was further enhanced in comparison with those cells cultured on pure CNF and TCPS. Moreover, as shown in Figs 9 and 10, the contact culture manner could apparently enhance the osteogenic differentiation of BMSCs more efficiently than the non-contact culture manner. It had been reported that carbon nanomaterials had the ability to promote osteogenic differentiation benefiting from their electric property^43^. In the osteo-inductive culture medium, bioactive components including dexamethasone, β-glycerophosphate disodium and ascorbic acid were added. Carbon nanomaterials had demonstrated ability in absorbing these bioactive components. Dexamethasone had aromatic structure which could interact with the graphite structure of carbon material by π-π bond^44^. Thus, the absorbed dexamethasone on CNF could promote the differentiation of BMSC and mineralization. Ascorbic acid and β-glycerophosphate could also be absorbed by CNF to exert a synergy with dexamethasone to increase the ALP activity of BMSCs^45^. On CNF/BG hybrids, therefore, the combination of CNF and osteo-inductive BG components showed significant synergistic promotion in osteogenic differentiation of BMSCs. On CNF/BG(68 s), which provided the most abundant silicium ions, the osteogenic differentiation of BMSCs was highly promoted.

The signaling pathways relating to the osteogenic differentiation of BMSCs were complex because there were many branches and crosses between different pathways^46^,^47^. Among them, BMP signaling pathway was studied intensively and noggin was found a specific inhibitor of BMP activity^48^. In this study, the effect of CNF/BG hybrids on the osteogenic differentiation of BMSCs was a comprehensive result of many cues from the materials. To identify if BMP signaling pathway being involved in the osteogenic differentiation of BMSCs on CNF/BG hybrids, noggin was applied in the culture of BMSCs on CNF/BG(68 s). As shown in Fig. 11, the expressions of the six osteogenic differentiation related genes all decreased significantly when the BMP activity was blocked by inhibitor noggin. Reasonably, it was suggested that BMP signaling pathway should be one essential pathway in determining osteogenic differentiation when BMSCs interacted with CNF/BG hybrids.

In summary, CNF/BG hybrids were readily prepared by incorporating BG component into CNF via combination of sol-gel method, electrospinning technique and thermal treatment. They were proven promising materials for bone repairing due to their biocompatibility, cell affinity and ability in inducing osteogenic differentiation of BMSCs. In comparison with pure CNF, the increasing surface roughness of CNF/BG hybrids, as well as the ionic dissolution products from CNF/BG hybrids, demonstrated a synergistic effect in promoting the proliferation and osteogenic differentiation of BMSCs. However, the contact effect would play a more important role than the soluble ions in regulating cell biological behaviors. By down-regulating the BMP activity, the fact that the expressions of osteogenic differentiation related genes were reduced, implied BMP signaling pathway to be one possible mechanism for CNF/BG hybrids exerting their function in inducing osteogenic differentiation of BMSCs.

## Methods

### Materials

PAN (M_w_ = 100,000 g/mol), containing 93.0 wt.% acrylonitrile, 5.3 wt.% methylacrylate and 1.7 wt.% itaconic acid, was purchased from Courtaulds Co. (UK). BG precursors including triethyl phosphate (TEP), calcium nitrate tetrahydrate (CN) and tetraethoxysilane (TEOS) were purchased from Aldrich (USA) and used directly. N,N-dimethylformamide (DMF, 99.5%) was bought from Tianjin Fine Chemical Co. (China). Other reagents required for the experiments were of analytically pure grade and obtained from Beijing Fine Chemical Co. (China).

### Preparation of CNF/BG hybrids

CNF/BG hybrids were prepared similarly to our previous work^21^. Briefly, TEP was dissolved in a mixture of aqueous ammonia, deionized water and ethanol (volume ratio being 1:3:3), followed by being stirred 24 h at 80 °C to complete the hydrolysis of TEP. Then CN and TEOS were added into the hydrolyzed TEP solution and the system was stirred at room temperature for 5 days to form BG precursor sol-gel. A certain amount of the sol-gel solution was added into PAN solution (10 wt.%) in DMF, followed by being stirred 12 h at room temperature to form a homogenous solution. The feeding ratios of each ingredient in preparing various CNF/BG hybrids are listed in Table 1. Subsequently, the solutions were electrospun at a flow rate of 0.4 ml/h in an electric field with the voltage of 15 kV. The produced nanofibers were received by a rolling rod with a surface linear rate of 12 m/s, which was placed at 15 cm away from the needle tip. The as-spun nanofibers were hydrolyzed at 50 °C for 24 h, stabilized at 280 °C for 2 h in air and carbonized at 1000 °C for 1 h in N_2_ atmosphere. Pure CNF was prepared similarly by using the same electrospinning and thermal treatment parameters. For clarity, the prepared CNF/BG hybrids were termed as CNF/BG(49 s), CNF/BG(68 s) and CNF/BG(86 s), respectively, according to their initial feeding doses of different precursors.

### Characterizations

Morphology of CNF/BG hybrids was observed by scanning electron microscope (SEM, Zeiss Supra 55, Germany) at an accelerating voltage of 20 kV. Before the observation, samples were sputter-coated with gold for 30 s by a sputter coater (Polaron E5600, USA). Compositions of CNF/BG hybrids were analyzed by element mapping and energy-dispersive spectrometry (EDS), which were performed similarly to SEM observation with exposure time of 180 s. The distribution of BG nanoparticles in CNF/BG hybrids was observed by using transmission electron microscope (TEM, JEOL 2000 EX) at an accelerating voltage of 200 kV. The contents of BG component in CNF/BG hybrids were determined by themogravimetric analysis (TGA, TA, Q-50) from room temperature to 800 °C in air atmosphere at a heating rate of 20 °C/min.

### Ion release from CNF/BG hybrids

CNF/BG hybrids (20 mg) were immersed in deionized water (8 ml) at 37 °C under continuous shaking (50 rpm) for a period of 15 days. To mimic the cell culture condition, the release medium was firstly refreshed at day 1 and then refreshed every other day. At each time point, release media were collected, and the concentrations of Ca^2+^, SiO_3_^2−^ and PO_4_^3−^ in the media were identified with an inductively coupled plasma optical emission spectrometer (ICP-OES, Optima 5300 DV, Perkin Elmer, USA). Then ion release curves were plotted as function of the immersing time. For each CNF/BG hybrid, the ion release experiment was performed three times for averaging.

### Cell culture

Sprague-Dawley rat BMSCs were purchased from Cyagen Biosciences (Guangzhou, China), which had been transfected by GFP. Cells were maintained in a complete Dulbecco’s Modified Eagle’s Medium (DMEM) supplemented with 10% fetal bovine serum (FBS, Gibco, USA), 100 IU/ml penicillin (Sigma, USA) and 100 mg/ml streptomycin (Sigma, USA). BMSCs were cultured in an incubator (Sanyo, Japan) with 5% CO_2_ supply at 37 °C and saturated humidity. Once reaching 80% confluence, the cells were digested by 0.25% trypsin (Sigma) and 0.02% ethylene diamine tetraacetic acid (EDTA) for further use. For osteogenic differentiation assay, 0.05 mmol/L vitamin C (Sigma, USA), 10 mmol/L β-sodium glycerophosphate (Sigma, USA) and 1 × 10^−8^ mol/L dexamethasone (Sigma, USA) were added to the culture medium. The culture medium and the osteogenic inductive medium were refreshed every two days.

The CNF/BG hybrids and pure CNF used for cell culture were cut into circular patches and placed into culture plates. Before cells were seeded, the substrates were immersed in 75% ethanol with exposure to ultraviolet (UV) light for 2 h, followed by three times phosphate buffered saline (PBS) washing and being immersed in DMEM overnight. Then, BMSCs would be seeded and cultured with the addition of culture medium or osteogenic inductive medium.

### Cell viability assay

Cell viability was evaluated with Alamar blue assay in two culture manners. Patches of CNF/BG hybrids and pure CNF sheets (ϕ = 6 mm) were placed into 96-well culture plates (Corning, USA) or 24-well Transwell (0.4 μm, Corning, USA). In the former case, the cells were seeded onto the materials directly (i.e. the contact manner), while in the latter case, the cells did not touch the materials (i.e. the non-contact manner). In both cases, BMSCs cultured on tissue culture polystyrene (TCPS) were taken as the control. Then five hundred BMSCs were added into each well and cultured 1–7 days to evaluate cell proliferation. One day after cell seeding, the culture medium was taken away and the wells were rinsed one time with PBS. Subsequently, 100 μl of Alamar blue (Thermo, USA) solution (1:10 diluted from the Alamar blue stock solution with DMEM) was added into each well. After incubation at 37 °C for 2 h, fluorescence of the liquid was quantified at the excitation and emission wavelength of 530 nm and 590 nm, respectively, using Luciferase tester (Polarstar^™^, Australia). Because of the nontoxicity of Alamar blue at the suggested concentration, fresh DMEM containing 10% Alamar blue was re-supplied into the culture plates and the cells were cultured continuously. At day 3, 5 and 7 after cell seeding, Alamar blue analysis was applied similarly to determine cell proliferation. For each sample, three independent experiments were conducted for averaging.

### Cell morphology

At 1, 3, 5 and 7 days after BMSCs being seeded onto CNF/BG hybrids, cellular constructs were retrieved from culture plates for SEM observation. Briefly, cellular constructs were rinsed with PBS for three times, and then fixed by 2.5% glutaraldehyde (Beijing Chemical Plant, China). After the fixed specimens were dried naturally in air, they were scatter-coated with gold and observed by SEM. For fluorescent observation, the fixed specimens were further incubated with Hoechest 33258 to stain cell nuclei, and the transfected cells could self-luminous by GFP to present cell cytoskeleton. Then, fluorescent images were captured by using laser scanning confocal microscope (LSCM, Leica SP8).

### qRT-PCR assay

Patches (ϕ = 16 mm) of CNF/BG hybrids and pure CNF sheets were placed into 24-well culture plates or 24-well Transwells, and 1 × 10^4^ BMSCs were seeded into each well. TCPS was used as control. After 1 day culture, the culture medium was replaced by osteogenic inductive medium to induce osteogenic differentiation. Then at the 3, 7 and 14 days of cell culture, cellular constructs were retrieved and rinsed with PBS, followed by the addition of 1 ml Trizol RNA extract kit (Invitrogen, Carlsbad, CA) to extract the total RNA. To quantitatively analyze the expression of osteogenic differentiation related genes, 2 μg RNA was used in a reverse transcription reaction to synthesize the first strand complementary DNA (cDNA). Six genes, including bone morphogenetic protein 2 (BMP2), runt-related transcription factor 2 (Runx2), osteopontin (OPN), alkaline phosphatase (ALP), osteocalcin (OCN) and collagen I (Col I), were selected, and their specific primers were designed as listed in Table 2. Quantitative RT-PCR was carried out with a StepOnePlus™ Real-Time PCR System (Thermo Fisher, CA). Relative RNA expression was calculated using a ΔΔCt method by being normalized with house-keeping gene 18 S to drop the influence of cell number, and three independent experiment were performed for averaging.

### ELISA assay

Patches (ϕ = 16 mm) of CNF/BG hybrids and pure CNF sheets were placed into 24-well culture plates or 24-well Transwells, and 1 × 10^4^ BMSCs were seeded into each well. TCPS was used as control. After 1 day culture, the culture medium was replaced by osteogenic inductive medium to induce osteogenic differentiation. Then at the 3, 7 and 14 days of cell culture, cellular constructs were retrieved and rinsed with PBS three times. Cell lysates were obtained by adding 1% Triton X-100 and calcium deposition were dissolved by 1 mol/L HCl. ALP activity and calcium deposition were determined with corresponding ELISA kits (Cloud-Clone, USA) and collagen I content was determined with Col I ELISA kit (Chondrex, USA) following manufacturers’ protocols. These three osteogenic makers expression was calculated with total protein by using BCA protein quantitation kit to drop the influence of cell number, and three independent experiments for each material were performed for averaging.

### BMP signaling pathway

BMSCs were seeded onto CNF/BG hybrids and induced osteogenic differentiation similarly as aforementioned. However, noggin (a specific inhibitor of BMP) was added into the osteogenic inductive medium at the concentration of 1000 ng/ml for the purpose to explore the mechanism of CNF/BG hybrids affecting the osteogenic differentiation of BMSCs. Then at the day 3 of cell culture, cellular constructs were retrieved and rinsed with PBS. After the addition of 1 ml Trizol reagent (Invitrogen, Carlsbad, CA), qRT-PCR were done as the description above to measure the expressions of osteogenic genes including Runx2, BMP2, OPN, ALP, OCN and Col I.

### Statistical analysis

Data were expressed as mean ± standard deviation (SD). Three independent experiments were carried out and at least 3 samples per each test were taken for statistical analysis. Statistical analysis was made by Student’s t-test between two groups and the differences were considered to be significant for p ≤ 0.05 and highly significant for p ≤ 0.01.

## Additional Information

**How to cite this article**: Zhang, X.-R. *et al*. Cell studies of hybridized carbon nanofibers containing bioactive glass nanoparticles using bone mesenchymal stromal cells. *Sci. Rep.* **6**, 38685; doi: 10.1038/srep38685 (2016).

**Publisher's note:** Springer Nature remains neutral with regard to jurisdictional claims in published maps and institutional affiliations.

## Figures and Tables

**Table 1:** Feeding ratios of each ingredient in preparing CNF/BG hybrids.

Sample	CN/g	Hydrolyzed TEP/ml	TEOS/ml	PAN/g	DMF/ml
CNF/BG(49 s)	0.529	0.156	0.559	2	20
CNF/BG(68 s)	0.296	0.154	0.756	2	20
CNF/BG(86 s)	0.067	0.152	0.959	2	20

**Table 2:** The primers designed for selected genes relating to the osteogenic differentiation of BMSCs.

Gene symbol	Forward primer (5′-3′)	Reverse primer (5′-3′)
BMP2	GGAAAACTTCCCGACGCTTCT	CCTGCATTTGTTCCCGAAAA
Runx2	TCCAGACCAGCAGCACTCC	TCAGCGTCAACACCATCATTC
OPN	AATGAAGGGCCCTGAGC	GCCAGTTCTGCAAGGAAGC
ALP	TCCCAAAGGCTTCTTCTTGC	ATGGCCTCATCCATCTCCAC
OCN	TATGGCACCACCGTTTAGGG	CTGTGCCGTCCATACTTTCG
Col I	GCATGGCCAAGAAGACATCC	CCTCGGGTTTGGACGTCTC
18S	GTAACCCGTTGAACCCCATT	CCATCCAATCGGTAGTAGCG
